# Remote-control Interrogation, Testing, and Reprogramming of Cardiac Implantable Electronic Devices with Telehealth Protocol for the Management of Patients at Home: The DOMUM REMOTUS Study

**DOI:** 10.19102/icrm.2023.14024

**Published:** 2023-02-15

**Authors:** Esteban Martin Kloosterman, Jonathan Z. Rosman, Eric J. Berkowitz, Murray Rosenbaum, Zachary Wettenstein

**Affiliations:** ^1^Cardiac Arrhythmia Service, Boca Raton, FL, USA; ^2^Florida Atlantic University, Boca Raton, FL, USA

**Keywords:** Cardiac electronic implantable devices, remote control, remote interrogation, remote programming

## Abstract

Remote control (RC) of cardiac implantable electronic devices (CIEDs) has been tested as safe and effective in the magnetic resonance imaging space. We sought to evaluate RC applications of patients at home. RC of cardiac devices in patients’ homes is feasible, safe, and effective, with consistent patient satisfaction. Patients with CIEDs using the CareLink™ network (Medtronic, Minneapolis, MN, USA) participated in a pair of home RC sessions. A technician visited the patient’s house and set up a telehealth tablet and a programmer, which included inputting a session key enabling programmer access via a third-party host. The investigator video-conferenced with the patient and remotely controlled the programmer for device testing and data assessment, using a cellular hotspot for Internet connection. Reprogramming was performed as necessary. In all cases, an RC session legend was programmed in the device information field as a control. The patients then completed an experience questionnaire. One hundred fifty patients (99 pacemakers and 51 implantable cardioverter-defibrillators) completed 2 RC sessions, for 300 RC sessions in total. There were no complications or communication interruptions once the system communication proved stable after the first minute. In 26 sessions, initial communication was interrupted upon device interrogation, requiring communication to be re-established (which sometimes necessitated switching to an alternative carrier). Clinically driven parameter reprogramming was performed in 58 RC sessions (39%). Programming of notations concerning RC sessions was performed in all 300 sessions. The average duration of the RC sessions was 11 min. Patients’ satisfaction scored 4.5 out of 5 points. In conclusion, RC management of cardiac devices at patients’ homes is safe, effective, convenient, and associated with high patient satisfaction. This technology may prove very useful in a changing health care delivery system, especially amid the coronavirus disease 2019 pandemic.

## Introduction

The use of remote control (RC) technology to manage cardiac implantable electronic devices (CIEDs)—pacemakers and implantable cardioverter-defibrillators (ICDs)—has been tested before as safe and clinically effective in different settings, including the emergency room, cardiology offices, electrophysiology (EP) labs, and the magnetic resonance imaging (MRI) space.^1–9^ We hypothesized that RC applications could be used to bring CIED testing to patients at home, ultimately bringing medicine to them. This could have future positive implications in breaking down barriers to care and improving social determinants of health in various isolated populations.

Although the concept of home remote monitoring has been developed extensively over the past several years, the current models in place are not conceptually interactive and do not allow for device reprogramming or patient personalization at this time.

After approximately 2 years of conceptualization and planning for this study, the coronavirus disease 2019 (COVID-19) pandemic ensued. Upon beginning enrollment, it quickly became apparent that a new dimension could be added to the value and purpose of this study; at the time of this investigation, health care in general was making a massive shift toward the acceptance of virtual health care and telehealth modalities.

### Objectives

The objective of this study was to prove that RC management of CIEDs in patients at home is feasible, safe, effective, and an acceptable modality of care for them.

RC of CIEDs is a reality today; thus, the challenge at this point is not the availability of the technology itself but rather establishing acceptance and a model allowing for systematic clinical application of the technology and expansion on a larger scale.

Although not used in this study, tablet device programmers with Wi-Fi connectivity are currently in clinical use. However, they lack RC features at present, which is why separate video-conferencing tablets and programmers had to be used.

## Methods

This was a single-center, prospective, non-randomized format study. Rolling enrollment was used until the achievement of 2 RC sessions per patient, resulting in a total of 150 patients.

We enrolled patients with pacemakers and defibrillators able to be monitored using the Medtronic CareLink™ network as an inclusion criterion. The protocol involved 2 clinical follow-up sessions using the at-home video-conferencing and RC session modality. All patients signed an informed consent form before enrollment. The protocol and informed consent were evaluated, approved, and supervised by the designated institutional review board regulatory agency.

The timing between sessions was not pre-specified, but we aimed for it to be <12 months. The study RC sessions did not preclude patients to be seen in the office or hospital as deemed necessary in between the remote sessions. The patients included were a mix of new patients enrolled for postoperative follow-up and established patients with devices interested in trying a new modality of service.

### Remote-control session workflow

**Figure 1:**
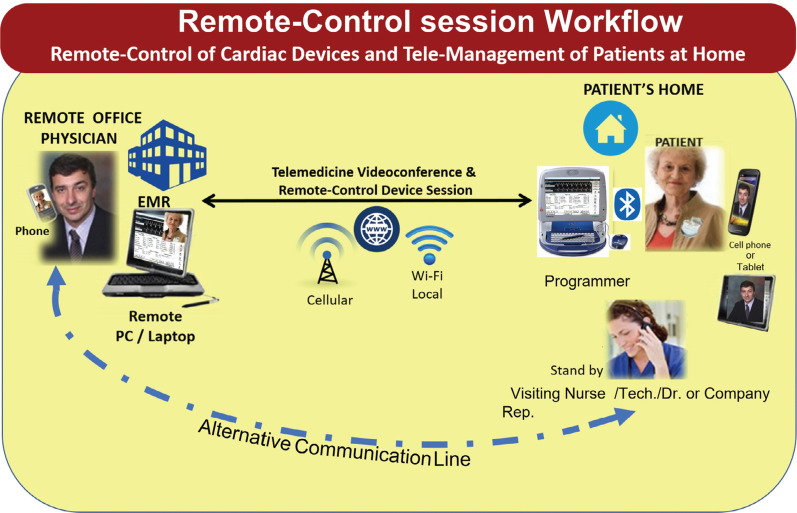
Workflow of the remote-control session.

**Figure 2:**
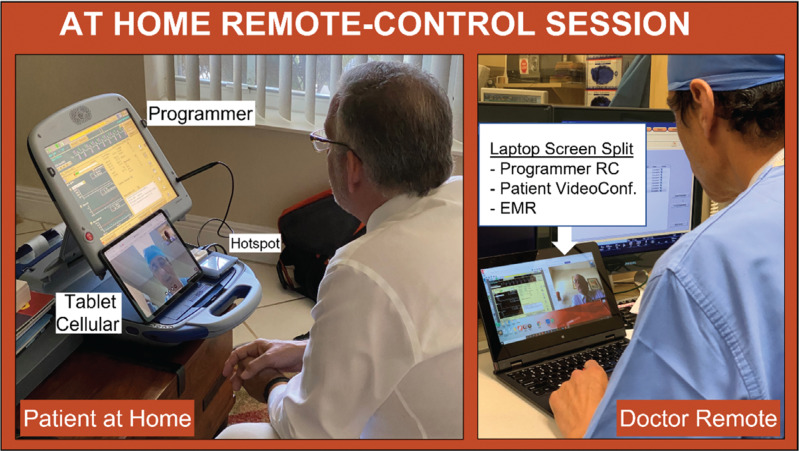
An example of the patient’s home and remote doctor’s setup of an RC session. On the patient’s side, one can see the programmer with RC connectivity, a hotspot connected to it, and a cellular tablet for videoconferencing. On the doctor’s side, the laptop displays the videoconference screen along with the RC programmer screen and EMR. *Abbreviations*: EMR, electronic medical record; RC, remote control.

A study technician visited each patient’s home and set up both a telehealth tablet (iPad; Apple, Cupertino, CA, USA) and a dedicated 2090 Medtronic programmer with a modem card and RC software REMOTECONTROL™ technology (see **Figure 1**). The study technicians were trained on the operation of the 2090 programmer and the protocol connectivity requirements. The technicians were not to participate in any intervention other than setting the system in place, ie, they were not to make any clinical judgment or decision. Resuscitation skills were not required. Of note, both technicians and patients were observant of the U.S. Centers for Disease Control and Prevention (CDC) COVID-19 recommendations and precautions for all encounters (see **Figure 2**).

The tablet used for patient interviewing and engagement was equipped with cellular connectivity provided by a single carrier (Verizon Communications, New York, NY, USA); alternatively, FaceTime (Apple) was used (via an iPhone [Apple] with AT&T [Dallas, TX, USA] as a carrier) if deemed necessary by the technician when they could not achieve adequate video or audio quality using the former setup. The programmer was connected to the Wi-Fi provided by the study hotspot. Two different carriers were available for optimal connectivity at all times. It should also be noted that, although the tablet could also connect to the hotspot Wi-Fi, it was decided to leave all the available bandwidth for the programmer to ensure the best transmission rates.

The patients had the liberty of choosing a place at home that they felt comfortable in, and, if necessary, the technician at times looked for areas of stronger and more adequate/stable cellular connection. On 3 occasions, as per the patient’s preference, the RC session was conducted outside the house in a patio area. The patients were positioned in such a way that they were facing the tablet for teleconference while also allowing the programmer to adequately visualize them so as to witness the session in real time. Patients were offered explanations and, in some cases if interested, shown findings as the session progressed.

The technician first logged in to a video-conference interview set up by the investigator. The investigator was already logged in to a third-party (Bomgar; BeyondTrust Corporation, Atlanta, GA, USA) communication website and would then generate a session key, which was a number randomly assigned by a Medtronic security software.

The technician was then instructed to input the key number to enable the programmer to have encrypted communication via the third-party host (Bomgar). At this point, the investigator had RC of the programmer for usual device function testing and data assessment. Of note, given previous experience with the system losing communication when the session was started remotely, it was decided for practical and consistency purposes that the technician would initiate the session after inputting the session key. The technician would have no other interaction with the programmer, although the technicians were trained and proficient to do so if instructed, as a safety backup.

**Figure 3:**
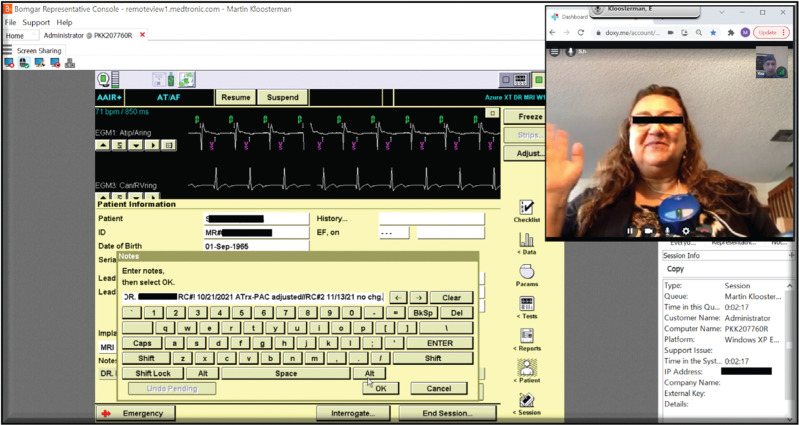
RC computer screenshot depicting the programmer as seen in the third-party RC site and the patient’s videoconference picture. The legend programming regarding the RC session documentation is shown. *Abbreviation*: RC, remote control.

At this stage, the remote investigator had free use of the programmer and parameters for reprogramming, which was performed as clinically deemed necessary. In all cases, a study mandatory legend regarding the RC session was entered in the comments section and programmed in the device information field as a control (see **Figure 3**).

The initial interrogation of the device provided the usual data, including device longevity, lead sensing, impedance, and automated thresholds as well as device usage, heart rate histograms, arrhythmic events, and alerts. As such, pacing thresholds were not routinely rechecked except in circumstances when it was felt clinically needed as well as to gather left ventricle testing vectors. Of note, if communication was interrupted during threshold testing, the programmer reacted in the same way as if bedside testing was being performed: the operator was to cease stylus contact with the screen, reverting to the baseline setting.

The REMOTECONTROL™ technology labeling restrictions did not allow its use for the following: underlying rhythm testing, cardioversion, electrophysiology studies (arrhythmia inductions), and programmer system analysis. As such, if it was thought clinically necessary to assess the underlying rhythm, we performed threshold testing in the VVI mode at 40 bpm with high output as a surrogate.

After their second visit, each patients filled out a survey questionnaire to gather feedback.

## Results

A total of 156 patients were enrolled; the first RC session was performed on May 1, 2020, and the second session was performed on February 10, 2022, for a total of 21 months of study duration. Of the 156 patients, 150 completed both sequential RC sessions and were included in the study for further analysis. Six patients (4 with pacemakers and 2 with ICDs) only completed a single RC session and therefore were not considered in the main analysis; 5 of them died of causes unrelated to the RC session before their second visit and 1 moved out of town after their first RC session.

The average interval between sessions was 94 days (range, 3–398 days) across a total of 300 RC sessions. The cardiac devices involved included 99 pacemakers and 51 ICDs (defibrillators) broken down into subgroups as follows: 92 DDD pacemakers, 1 AAI pacemaker, 1 leadless pacemaker (Micra™; Medtronic), 5 BIV pacemakers, 30 BIV-ICDs, 20 DDD-ICDs, and 1 VVI-ICD. Both postoperative and follow-up settings were assessed during the patient’s initial RC visit (see **Table 1**). The postoperative period was considered to last for <6 weeks (<42 days) after implantation, and 48 patients fell into this category. The other 102 patients were seen during standard device follow-up visits. The demographics of the 150 patients revealed a total of 96 men (64%) and 54 women (36%) with an average age of 77 years each.

There were no study-related complications, and no patient required an in-office visit in between the RC sessions due to a non-addressed clinical issue or direct complication of this modality. No rescue interventions were needed during RC sessions. At no time was there a need for study technician intervention other than in the context of their specified role. There was a communication interruption during 1 RC session, requiring a in of the Wi-Fi carrier while the session was ongoing; otherwise, there were no communication interruptions once the connection was proven stable after establishing adequate connectivity past the first minute. In 26 sessions, the initial remote device interrogation communication was interrupted upon device interrogation, requiring it to be re-established (sometimes resourcing to an alternative cellular carrier).

Two established cellular carriers (Verizon, n = 127; AT&T, n = 23) were available to be used during RC sessions. After setting up the programmer and the iPad, the study technician evaluated the signal strength of both carriers to decide which one to start the protocol with. It was decided for the purposes of this study that the “hotspot” network would be exclusively dedicated to the programmer connection, never adding, that is, the iPad connection. This was largely a precautionary decision that could be further explored in future studies and applications of use. The iPad connection had a dedicated Verizon cellular connection. Doximity (Doximity, Inc., San Francisco, CA, USA) was the program used to perform the telehealth interview. When no adequate bandwidth was available to run Doximity, FaceTime via iPhone with AT&T connectivity service was used instead. As a result, Doximity on the iPad was used in 118 and FaceTime was used in 32 sessions, respectively.

The RC sessions were conducted mainly from the local office but also were performed at other locations, including 2 different local hospital facilities; investigators’ homes (all in Boca Raton/Delray, FL, USA); and other locations in the United States (including in Miami, Orlando, and Nashville) and worldwide (in St. Martin, Peru, Uruguay, Greece, and Turkey). High-speed Internet was desirable; however, as long as the Internet connection was stable, there were no issues with international use of the system. No connectivity failures were experienced in any instance when the system was accessed from abroad.

The objective of these visits was to evaluate the patient’s clinical condition and subsequently optimize the device functionality according to the patient’s particular needs. Clinically driven parameter reprogramming was performed in 58 RC sessions (39%). The most common reprogramming changes were in pacing mode (mostly DDD/R to AAI/R-DDD/R), turning on or adjusting the rate response, adjusting the lower rate limit (mainly increasing to 60–70 bpm with the Sleep mode on at 60 bpm), and turning atrial therapies on (PAC response and AF ATP). There was no need to change ventricular tachycardia/ventricular fibrillation therapy programming in any of the cases. We did not encounter a clinical need to manually test thresholds in a pacer-dependent patient, but there were no restrictions on doing so.

Study mandatory programming of annotations regarding RC session details was performed in all of the encounters. The average duration of the RC sessions was 11 ± 3 min. This time included some additional study-related measures that would not be needed during routine clinical follow-ups, such as obtaining and archiving screenshots or accessing the information section of the device to program the study entry, for which routine clinical follow-ups would be expected to be somewhat shorter. Of note, telehealth images were captured in all encounters, including some wound check illustrations, in the postoperative follow-up cases.

A patient survey questionnaire was provided after the second RC session, and 129 of 150 patients (86%) completed the questionnaire (see **Table 2**). Responses revealed that 85% of the respondents felt “extremely confident” to “very confident” about establishing this model as part of their health care. The majority of patients (85%) felt comfortable with the session and would recommend it to friends, family, or other patients. The most common reservation was giving up personal interactions with the physician, and the patients requested that, if implicated, they would still have some “face-to-face” or in-person interactions at some point during the year. Compared to the standard system of an in-office check, 81% of the patients considered the RC session to have superior advantages. Almost all patients (98.5%) responded that they would recommend it to others. To the best of our knowledge, there were no negative comments about the RC sessions and no patient withdrawal from the study due to problems, difficulties, or dissatisfaction with this modality.

## Discussion

Study limitations include that this was a single-center study that was neither controlled nor randomized.

The REMOTECONTROL™ technology by Medtronic is an application approved by the U.S. Food and Drug Administration and currently used in clinical practice, mainly in MRI environments where company representatives log in remotely to the programmer to change the device settings to an MRI-safe mode and then back to baseline. We published the first study in this regard that led to its clinical use.^7^ With that said and with an understanding of the many concerns this new technology may bring, we want to point out that the system itself has already been validated and, as such, is not the specific focus of the present study; instead, the actual clinical application of the technology in the particular setting of a patient’s home is the primary subject of interest.

We chose to perform 2 RC sessions per patient to establish reproducibility and facilitate patients’ understanding of this methodology, expanding their experience in order to potentially obtain more candid and insightful feedback.

We used dedicated study “hotspots” for consistency and to avoid having to ask to use a patient’s personal Wi-Fi, which would cause us to have to depend on their availability, password, and other technological variables. This also allowed the study to be more applicable to a wider population that may not readily have access to high-speed Internet. It remains to be seen how the communication aspect of the visit would be affected, having used different households’ Internet connection setups, which vary in terms of carrier, dependability, speed, and bandwidth.

The study began enrollment at approximately the same time the COVID-19 pandemic caused nationwide and global lockdowns; this was a complete coincidence, as the study was not conceived based on this notion. With new rules for office visits and the expansion of general telehealth visits, this modality was probably better accepted; however, it should be pointed out that, for the first 6 months of the study, there was a reduction in available patient populations and a relative decrease in any kind of follow-up or medical interaction. Thereafter, the idea of this at-home service modality was embraced due to not having to come to the office, use masks, and be in contact with other patients in the waiting room (despite our office providing all resources for personal protection, social distancing, and sanitation in accordance with the relevant CDC guidelines at that time). Instead, interpersonal interaction was limited to a single technician, who also abided by all CDC guidelines and recommendations at that time.

Beyond the COVID-19 situation, the study was conceived on the belief that patients with CIEDs could be effectively cared for with this at-home, remote-controlled telehealth service. The notion of telehealth in many instances was considered a useful tool for dealing with patients in rural areas or living far from specialized care centers. This is, of course, one true advantage of this service modality; however, in our view, there are a significantly greater number of patients who would benefit from this service in large cosmopolitan cities where transportation for the elderly is significantly complex (ie, not just from home to the office but also within the office premises because of the need for parking, walking long hallways, taking elevators or stairs, accessing bathroom facilities, and securing drivers and aides). For those, on the other hand, who are able to sort these hurdles independently, there is still the value of time saved and the convenience of completing the appointment from the comfort of their home.

Eventual advancements in the patient’s interface, such as the addition of RC capabilities to today’s existing “tablet” programmers, will allow easier integrated patient self-driven interaction facilitating the use of this feature on a larger scale. The present use of iPad-based programmers does not allow access to RC.

Lastly, the notion of RC access of CIEDs tends to spark cybersecurity concerns, which in general are founded on a perception of vulnerability that may lead to potential hazardous events but not in true existing system flaws. In this regard, from conception to present, we seemed to accept with no issue the fact that CIED programmers from all companies are the only computers in the whole health care system able to be operated without a user identification number and password—a clear double standard. The RC methodology used in this study and presently conceived for clinical use has several layers of encryption, uses a proprietary randomly timed digital key that needs to be manually keyed in onsite and an independent third-party remote computer access with encrypted transmission, and has been vetted by the company with vulnerability testing. Two different usernames and passwords are required to obtain the digital key and access to a third-party RC company. The key generated by the doctor remotely was verbally communicated in the video-conference to the onsite study technician, leaving no digital footprint to be interfered with.

As such, we are confident in the cybersecurity measures in place to assure system operation safety. However, broader systematic use of this modality will require a reassessment and likely update of the present measures in place.

## Conclusion

RC management of cardiac devices in patients’ homes is safe and effective, with high patient satisfaction and expansive convenience, particularly in times of the COVID-19 pandemic.

Imminent advancements in the programmer interface, leading it to become a patient self-driven tablet, will facilitate the use of this feature.

The designing of a systematic clinical use of this service model on a large scale will be the next challenge for health care systems, providers, and health insurance carriers.

## Figures and Tables

**Table 1:** Patient and Study-related Demographics

Patients	
Sex	
Male	96 patients
Female	54 patients
Age, years^a^	
<50	2 patients
50–70	48 patients
>70	130 patients
**Devices** ^b^	
DDD pacemaker	92 patients
BIV pacemaker	5 patients
BIV-ICD	30 patients
DDD-ICD	20 patients
VVI-ICD	1 patient
Other (AAI and Micra)	2 patients
**Cellular network**	
Verizon (primary)	127 sessions
AT&T (secondary)	23 sessions
**Communication app**	
oximity (primary)	118 sessions
FaceTime (secondary)	32 sessions

*Abbreviation:* ICD, implantable cardioverter-defibrillator. ^a^Average age, 77 years. ^b^There were 99 pacemakers and 51 implantable cardioverter-defibrillators total.

**Table 2:** Patient Survey Results (n = 129) Showing Largely Positive Responses to Study-related Procedures

Questions	Patient Responses				
	Extremely Confident	Very Confident	Adequately Confident	Somewhat Confident	Not Confident
Q1. Confidence in care and professionalism	81.50%	17%	1.50%	—	—
Q2. Confidence in continued use of this model	60%	25%	8.50%	1.50%	5%
	**Very Superior**	**Somewhat Superior**	**Equal**	**Somewhat Inferior**	**Inferior**
Q3. Remote system in comparison to in-person care	60%	21%	8.50%	3.50%	1.50%
	**Yes**	**Yes, with Reservations**			**No**
Q4. Recommendation of use to others	88.50%		10%		1.50%
Overall response rate	85% (129/150)				
